# Ventilation and features of the lung environment dynamically alter modeled intrapulmonary aerosol exposure from inhaled electronic cigarettes

**DOI:** 10.1038/s41598-024-81066-x

**Published:** 2024-12-30

**Authors:** Liqiao Li, Haoxuan Chen, Yifang Zhu, Airi Harui, Michael D. Roth

**Affiliations:** 1 Department of Environmental Health Sciences, Jonathan and Karin Fielding School of Public Health, University of California, Los Angeles, CA 90095-1772 USA; 2 Division of Pulmonary and Critical Care, Department of Medicine, David Geffen School of Medicine, University of California, Los Angeles, CA 90095-1690 USA

**Keywords:** Artificial lung, Inhalation exposure, Electronic cigarettes, Aerosol transformation, Respiration, Risk factors

## Abstract

Electronic cigarettes (e-cigs) fundamentally differ from tobacco cigarettes in their generation of liquid-based aerosols. Investigating how e-cig aerosols behave when inhaled into the dynamic environment of the lung is important for understanding vaping-related exposure and toxicity. A ventilated artificial lung model was developed to replicate the ventilatory and environmental features of the human lung and study their impact on the characteristics of inhaled e-cig aerosols from simulated vaping scenarios. Compared to static conditions, normal breathing decreased peak particle number concentrations (PNCs) and area under the curve (AUC) by 40% and 70%, respectively, and increased particle decay rates fourfold. However, even with ventilation, intrapulmonary PNC levels exceeded 2 × 10^6^ particles/mL in a 4-puff vaping session. Both respiratory rate and tidal volume modulated e-cig aerosol exposure in a manner inversely proportional to minute ventilation. The modeled lung environment (37 °C, 88% relative humidity) also significantly altered particle size distributions by facilitating aerosol transformations such as hygroscopic growth, which further impacted e-cig aerosol exposure and particle removal. This work highlights the dynamic nature of intrapulmonary exposures and underscores the need to account for lung physiology and environmental factors when assessing inhaled e-cig aerosols.

## Introduction

Electronic cigarette (e-cig) aerosols, produced through the vaporization of e-liquids, are fundamentally different from the tobacco smoke generated by the combustion of conventional cigarettes^1–3^. This distinction, coupled with appealing flavorings and aggressive marketing, has led to a surge in e-cig usage, especially among adolescents and young adults^4,5^. While existing studies generally agree that e-cig aerosols contain lower levels of toxic chemicals than tobacco smoke^6,7^, there is still limited data regarding the intrapulmonary exposure to e-cig aerosols from vaping, which is crucial for understanding their associated inhalational toxicity^8–10^. E-cig aerosols contain the same constituents as the e-liquids, including propylene glycol (PG), vegetable glycerin (VG), nicotine, flavorings and other additives, along with an array of volatile thermal degradation products generated during the heating/vaporization process^1–3^. Consequently, e-cig aerosols have unique physicochemical characteristics and are influenced by environmental conditions. For example, our previous studies have shown that e-cig aerosols are highly volatile and quickly evaporate in indoor environments, leading to a reduction in the size of e-cig particles^11,12^. Furthermore, e-cig aerosols can be highly dynamic in response to factors that include humidity, temperature, and dilution due to their impact on particle condensation, hygroscopic growth, and coagulation^10^.

Available research has typically focused on the emission profiles and characteristics of e-cig aerosols in experimental chambers or laboratory rooms under room temperature and humidity^13–16^. However, considering the distinct properties of e-cig aerosols, it is not clear that resulting data accurately reflect the nature of inhaled e-cig aerosols as they exist within the human lung. For example, the variable dilution ratios (i.e., the chamber volume divided by puff volume) used in laboratory settings can substantially alter the measured particle size distribution^17^. Previous studies have reported that the count median diameter (CMD) of e-cig particles can range from 18 to 386 nm when measured across a broad range of dilution ratios^18–23^. Additionally, the dynamic effects of the ventilatory process and lung environment are often overlooked in current lab-based practices. During vaping, a small puff of concentrated e-cig aerosols (e.g., 20–50 mL) is admixed with a larger full breath and then rapidly humidified and warmed as it is drawn through the oropharynx^10^. The inhaled breath distributes and further dilutes the e-cig aerosols throughout the total lung volume. After inhaling one puff, ongoing ventilation serially mixes, dilutes and exhales particles that remain suspended within the airways and alveolar regions. As such, the exposure of e-cig aerosols within the human lungs is a dynamic process and its assessment in vivo is further complicated by person-to-person variations in terms of vaping topography, lung size, and breathing pattern^24,25^. While several in silico studies have integrated aerosol dynamics into their computational modeling to predict aerosol behaviors in the human lung^23,26–30^, there is still a lack of experimental data to validate these results.

To address these knowledge gaps, we employed a ventilated artificial lung system to characterize inhaled e-cig aerosols in situ within a modeled lung chamber that simulates the overall volumes, breathing patterns, and environmental conditions (temperature and humidity) of human lungs. A 4^th^generation e-cig, the Virginia Tobacco-flavored JUUL pod, containing 5% nicotine salt with a PG/VG ratio of 30/70^31–33^, was used to generate the e-cig aerosols that were inhaled into the lung chamber. The outcomes of this work are the exposure profiles of e-cig aerosols, including real-time particle number concentrations (PNCs), and number- and mass-based particle size distributions, occurring in the lung chamber over a 4-puff vaping cycle that simulates a real-life usage pattern.

## Materials and methods

### The ventilated artificial lung system

**Fig. 1:**
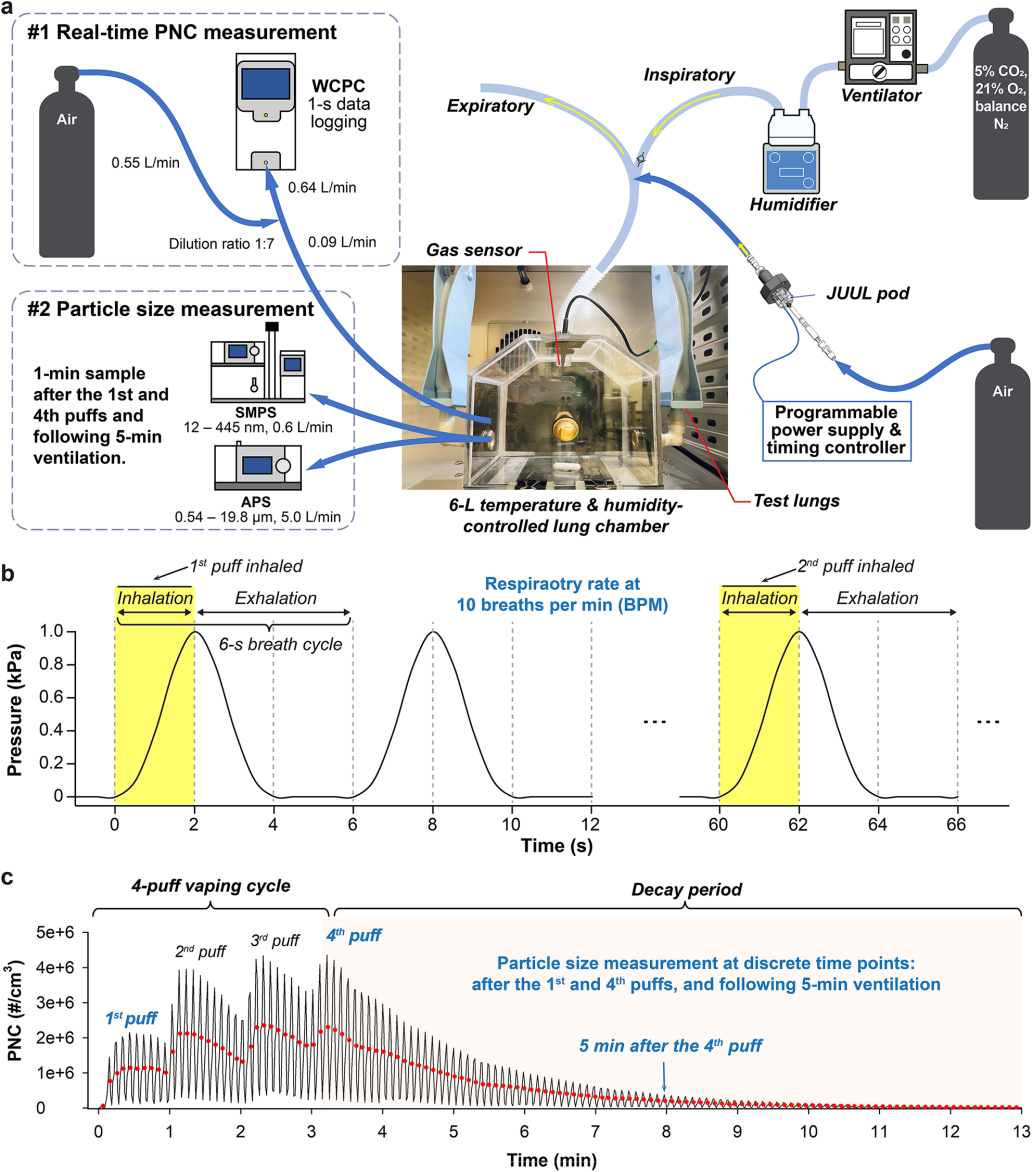
Ventilated artificial lung system and vaping exposure paradigm. (**a)** The central component is a temperature-controlled and pressure-regulated artificial lung chamber (6-L volume) connected to a ventilator with independent controls for inspiratory time, respiratory rate, tidal volume, heating and humidification. A JUUL-pod holder with a regulated flow of filtered air and a programmable power supply is used to generate e-cig aerosols in individual puffs in coordination with the ventilator. (**b**) A normal resting breathing pattern was established by setting the ventilator to 10 breaths/min (BPM), tidal volume of 480 mL, and inspiratory time of 2 s, which created a 6 s respiratory cycle (2 s inhalation; 4 s exhalation). At the initiation of a vaping session, a puff of e-cig aerosol is delivered into the inspiratory limb at the end of exhalation, and inhaled into the lung chamber by the next inhalation. Dynamic pressure measurements within the lung exposure chamber document these repetitive breathing and vaping cycles. (**c)** Particle number concentrations (PNCs) within the lung chamber over a 4-puff vaping session were measured in real time via the integrated sampling port. The lung chamber was continuously ventilated and 4 puffs of e-cig aerosols were delivered at 1-min intervals followed by a decay period for 10–12 min driven by the continued ventilation. A saw-tooth pattern (rise/fall) of recorded PNCs occurs with each breath due to the impact of symmetric inspiratory and expiratory pressure changes on flow rates to the water-based condensation particle counter (WCPC). Mean values () represent actual diluted PNCs. The particle size distributions were measured using a Scanning Mobility Particle Sizer (SMPS) and Aerodynamic Particle Sizer (APS) at discrete time points: after the 1^st^ and 4^th^ puffs and following 5 min of ongoing ventilation.

A ventilated artificial lung system was developed from three major components (Fig. 1a**)**: 1) a temperature-controlled and pressure-regulated lung chamber; 2) a volume-controlled ventilator (EPV100, Allied Healthcare Inc., USA) with integrated heating and humidification (MR730, Fisher & Paykel Healthcare Ltd., New Zealand) and a single limb ventilator circuit (Model L599-130, Allied Healthcare Products Inc., USA); and 3) a programmable e-cig puffing apparatus. Inert polycarbonate sheet material was fabricated into a gas-tight exposure chamber that approximates the conical shape and average lung volume (6 L) of an adult male (47-year-old, 185 cm high, and 80 kg) at the end of inhalation, calculated as the functional residual capacity (FRC) plus 50% of the inspiratory capacity (IC) according to the Global Lung Function Initiative (GLI) calculator^34^. Two elastic test balloons (Venti Plus, Maxtec LLC., USA) were symmetrically attached to each side of the lung chamber, expanding during inhalation and then contracting to ‘exhale’ by their elasticity, mimicking lung volume changes during breathing (e.g., tidal volume). Their compliance was specifically tuned to regulate intrapulmonary pressures within normal ranges. Sensing probes were integrated to monitor relative humidity (RH), temperature, and pressure. This artificial lung chamber was housed within a controlled climate chamber equipped with video recording. Integrated sampling ports allowed for in situ aerosol sampling and measurement.

Active ventilation (breathing) was generated by the ventilator, delivering breaths of a pressurized gas mixture to replicate the native lung environment (5% CO_2_, 21% O_2_, balance N_2_) with independent controls for inspiratory time (set at 2 s), tidal volume (200 to 1200 mL) and respiratory rate (8–20 breaths per minute [BPM]). A single limb ventilator circuit directed each breath into the lung chamber with a one-way valve that directed expiratory flow through a separate low-resistance exhalation port. Simulated normal breathing at rest was set at 10 BPM, to simplify the operational setup of our system, along with 480 mL/breath (6 mL/kg). These parameters created a 6-s respiratory cycle (2-s inhalation; 4-s exhalation) with a resting minute ventilation of 4.8 L. Respiratory cycles were monitored by the air pressure changes within the lung chamber (Fig. 1b**)**. By independently controlling temperature and RH using the in-line humidifier and a climate chamber, we created three environmental conditions for comparison including a dry environment (3 ± 3% RH, 25 ± 0.5 °C), a room environment (38 ± 3% RH, 25 ± 0.5 °C), and a simulated lung environment (88 ± 5% RH, 37 ± 0.5 °C). The chamber was flushed with dry filtered air between tests to clear particulates, heat and humidity.

A Virginia Tobacco-flavored JUUL pod containing 5% nicotine salt with a PG/VG ratio of 30/70 was used for testing in this study^31–33^. The e-cig puffing apparatus was composed of a HEPA-filtered compressed air source, a JUUL pod holder (Automate Scientific Inc., USA), a programmable controlling board and a DC supply to operate the e-cig device at 3.7 V^12,35^. To coordinate e-cig puffing with the ventilator cycle, e-cig aerosols were generated (1 L/min flow rate; 2 s puff duration) during the final 2 s of a breath cycle and delivered to the inspiratory limb of the ventilator circuit immediately prior to the next inhalation. This created a 33 mL e-cig aerosol puff volume, consistent with the reported vaping topographies for the fourth generation of e-cigs, which tend to have a smaller puff volume due to their high nicotine salt concentrations^36,37^. As shown in Fig. 1b, the entire puff volume was then delivered into the lung chamber by the subsequent inhalation. A control valve between the e-cig apparatus and inspiratory limb was closed after each puff. In this study, a typical vaping session was established with a 4-puff vaping cycle at 1-min intervals over 3 min followed by a 10-min decay period in which ongoing breathing gradually diluted and washed e-cig aerosols out of the lung chamber. As shown in Fig. 1c**,** the PNCs measured in real time over a typical vaping session demonstrated a progressive accumulation of e-cig particles within the lung chamber after each puff (of the 4-puff vaping session), as well as the gradual removal of inhaled particles between the puffing intervals and after the last puff. In the presence of ventilation, the cyclical pressure changes within the chamber created a fluctuating pattern of real-time PNC readings (reflecting pressure-induced changes in flow rate with each breath). Control experiments established that the mean PNC, between each peak and valley, corresponded with the running average chamber PNC (dots in Fig. 1c). Experimental figures and data analyses were generated using these mean values.

To understand the impact of ventilation on inhaled particles, a static condition was also studied using the same artificial lung system with the following modifications: Only one breath was administered from the ventilator with each puff in order to deliver the e-cig aerosol into the lung. The ventilator was turned off between vaping puffs and after the 4^th^ puff (during the decay period). A magnetic stirrer was used to maintain the suspension and mixing of the e-cig aerosols within the chamber.

### Aerosol measurements and characterizations

PNCs and particle size distributions were measured separately (Fig. 1a). Given that e-cigs predominantly emit submicron and ultrafine particles which dominate particle number concentrations, we employed real-time PNC monitoring using a water-based Condensation Particle Counter (WCPC 3788, TSI, Inc., USA) with a data logging interval of 1 s. This allowed us to accurately map the dynamic exposure to e-cig aerosols throughout the vaping session. A dilution with HEPA-filtered air at a ratio of 1:7 was used to ensure that the measured PNCs fell within the measuring range of the device, and the measured PNCs were therefore corrected for this dilution factor. As detailed in Table 1, real-time PNCs were measured within the ventilated or static lung chamber (ventilator on or off, respectively), under three different environmental conditions, and with three different breathing patterns. At least three independent experiments were conducted for each experimental condition.

**Table 1:** Summary of experimental conditions.

Lung model	Environmental conditions	Breathing patterns	Data Type collected
Ventilated	Dry Environment (3 ± 3% RH, 25 ± 0.5 °C)	10 BPM, 480 mL/breath	Particle size distribution
	Room Environment (38 ± 3% RH, 25 ± 0.5 °C)	10 BPM, 480 mL/breath	PNC, particle size distribution
		10 BPM, 720 mL/breath	PNC
		15 BPM, 720 mL/breath	PNC
	Lung Environment (88 ± 5% RH, 37 ± 0.5 °C)	10 BPM, 480 mL/breath	PNC, particle size distribution
		10 BPM, 720 mL/breath	PNC
		15 BPM, 720 mL/breath	PNC
Static	Dry Environment (3 ± 3% RH, 25 ± 0.5 °C)	N/A	Particle size distribution
	Room Environment (38 ± 3% RH, 25 ± 0.5 °C)		PNC, particle size distribution
	Lung Environment (88 ± 5% RH, 37 ± 0.5 °C)		Particle size distribution

PNC = particle number concentration, BPM = breaths per min, RH = relative humidity.

To characterize the particle size distributions of inhaled e-cig aerosols, key factors determining their exposure through deposition, we employed a Scanning Mobility Particle Sizer (SMPS 3080, TSI, Inc., USA) and an Aerodynamic Particle Sizer (APS 3321, TSI, Inc., USA) to measure e-cig aerosols from the lung chamber at three discrete time points: after the first and last vaping puff, and following 5 min of ongoing ventilation (Fig. 1c). Their measuring modes were optimized to measure size ranges of 12 – 445 nm (electric mobility diameter by SMPS) and 0.54 – 19.8 µm (aerodynamic diameter by APS), covering most e-cig emissions. When measuring the particle size distribution, ongoing ventilation was turned off at the specified time point to create a static environment within the chamber for sample collection. As such, only one measurement was conducted from each experiment in order to create accurate “snap shots” of the particle size distribution at a specific time point. Six conditions were evaluated including the dry, room, and simulated lung environments with each of these measured under both ventilating and static chamber conditions (Table 1). Triplicate experiments were carried out for each condition and at each time point. The internal video system allowed qualitative visualizations of the e-cig aerosols within the lung chamber and comparative images captured immediately after the fourth puff for each of the six experimental conditions.

### Data and statistical analysis

The peak PNCs and area under the curve (AUC) resulting from each vaping puff and over the entire vaping session were obtained from the real-time PNC curves. A regression of the first-order decay was used to determine the particle decay rates (k) over the 10-min decay period of the vaping sessions^12^. E-cig aerosols within the lung chamber were considered well mixed due to the continuous breathing (ventilated condition) or the presence of a magnetic stirrer (static condition). The differences in peak PNCs, AUC, and decay rates among experimental conditions were examined using the One-way ANOVA with pairwise comparisons. p < 0.05 was considered significant.

The size distribution data measured by SMPS and APS were matched for the same test and averaged from independent tests for each experimental condition using Data Merge software (TSI, Inc., USA, version 1.1, https://tsi.com/product-accessories/data-merge-software-module-390069). The mobility particle diameter measured by the SMPS was converted to the aerodynamic particle diameter, and the mass-based particle size distributions were calculated from the directly measured number-based particle size distributions by SMPS and APS, assuming spherical particles with a density equivalent to that of the parent e-liquid (1.15 g/cm^3^)^31^. In this study, no aerosol modes (number-based) fell into the size gap between the SMPS and the APS. Each aerosol mode was fitted to a lognormal or Rosin–Rammler distribution, according to the best fit, and then summary statistics for each mode were calculated including the median, mean, geometric mean (GM), geometric standard deviation (GSD), and total number and mass concentrations. The time-resolved particle size distributions were plotted with the size range gap between SMPS and APS output (484 – 540 nm, aerodynamic diameter) and time gaps between three discrete time points (i.e., t = after first and fourth puffs and following 5 min of ongoing ventilation) fitted by linear interpolation.

The statistical analysis and visualization of the results were performed using Sigmaplot (Systat Software, Inc., USA, version 14.0) and Prism (GraphPad Software, Inc., USA, version 9.0).

## Results

### Effects of ventilation on e-cig aerosol exposures within the artificial lung chamber

**Fig. 2:**
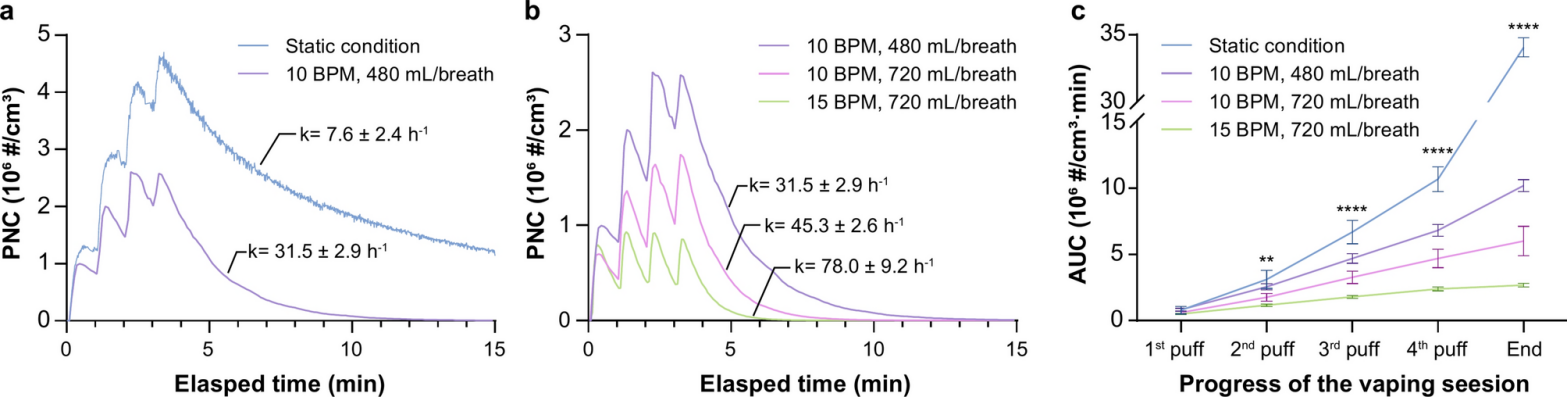
Exposure profiles of e-cig aerosols in the artificial lung chamber during a simulated 4-puff vaping session, in which 4 puffs of e-cig aerosols were delivered into the lung chamber in a room environment (38 ± 3% RH; 25 ± 0.5 °C) at 1-min intervals and followed by a 10-min decay after the last puff. (**a, b)** Real-time PNCs within the artificial lung chamber under the static and various ventilated conditions. The PNC curves are representative examples for each experimental condition, and the labeled decay rates (k) are presented in 95% CI from the triplicate experiments; (**c)** Area under the real-time PNCs curve (AUC) over the simulated vaping session. The error bars stand for standard deviations from triplicate experiments. Significant differences in AUC among different ventilation patterns at each time point were analyzed via One-way ANOVA (*: p < 0.05, **: p < 0.01, ***: p < 0.001, ****: p < 0.0001).

Lung exposure to inhaled e-cig aerosols is a dynamic process and the model employed in our study simulates the generation of a concentrated puff of e-cig aerosols, its mixing with the inhaled breath, subsequent dilution within the greater lung volume and gradual removal of inhaled particles through ongoing breathing. A 4-puff vaping cycle, with a 33 ml puff volume and a 1-min puff interval, was studied as an estimate of a realistic average vaping session for users of a 4^th^generation pod device^38,39^. Initial studies were performed in a standard room environment (38% RH, 25 °C) to be consistent with standard laboratory practices. As shown in Fig. 2a, the peak PNCs within the lung chamber increased in a step-wise manner as each additional puff of aerosols was introduced, regardless of the presence or absence of ongoing ventilation. However, while the peak PNC after the 4^th^ puff reached 4.32 ± 0.91 × 10^6^ #/cm^3^ (95% CI) under static conditions, it only reached 2.61 ± 0.64 × 10^6^ #/cm^3^ (95% CI) in the presence of ventilation at a resting breathing pattern (10 BPM and 480 mL/breath). This direct comparison indicated an average 40% reduction in the peak PNCs primarily due to the serial dilution and exhalation of particles from ongoing breathing during the puffing intervals. Ongoing breathing also substantially impacts on the decay rate of particles after the last e-cig puff, leading to a much faster decay rate (31.5 ± 2.9 h^−1^, 95% CI) as compared to the static condition (7.6 ± 2.4 h^−1^, 95% CI). As a result, the introduction of a resting breathing pattern led to lower intrapulmonary exposures compared to those under the static condition with an overall 70% reduction in the AUC for PNCs within the lung chamber (Fig. 2c). Increasing the minute ventilation (tidal volume × respiratory rate), by serially changing respiratory rates (10 or 15 BPM) and tidal volumes (480 or 720 mL) resulted in a proportional decrease in intrapulmonary exposures to e-cig aerosols (Fig. 2b**)**. When the respiratory rate was held constant (10 BPM) but the tidal volume increased by 50% (from 480 to 720 mL/breath), the peak PNC also decreased by approximately 25% (1.96 ± 0.67 × 10^6^ #/cm^3^, 95% CI) and the particle decay rate increased approximately 1.44-fold (45.3 ± 2.6 h^−1^, 95% CI). When the tidal volume was then held constant at 720 mL/breath and the respiratory rate increased by 50% (from 10 to 15 BPM), the peak PNC decreased by another 49% (9.55 ± 0.21 × 10^5^ #/cm^3^, 95% CI) and the decay rate increased another 1.72-fold (78.0 ± 9.2 h^−1^, 95% CI). The minute ventilation was therefore highly correlated with both the peak PNCs and decay rates (r^2^ = 0.96 and ≥ 0.97, respectively) but in an inverse manner, as shown in **Fig. S1**. Consequently, ventilation resulted in fewer accumulated PNCs over time in the simulated vaping session, as indicated by the area under the curve (AUC) of the PNCs in Fig. 2c. The differences in AUCs between the static and various ventilation conditions increased over the progress of the vaping session and reached the largest when the entire vaping session was completed. Similarly, the AUCs for the entire vaping session under different ventilation conditions were directly correlated with the MV (r^2^ = 0.87), as presented in **Fig. S1a**. In addition, as the minute ventilation increased, we observed a plateau in the peak PNCs occurring at progressively earlier timepoints, eventually resulting in a plateau in the peak PNC after just 2 e-cig puffs. Overall, this model demonstrates the impact of continuous ventilation on maximal intrapulmonary exposure during a realistic vaping session, which replicates the established effects of changing minute ventilation on the removal of inhaled particles from the lung^40^.

### Effects of the lung environment on e-cig aerosol exposures within the artificial lung chamber

**Fig. 3:**
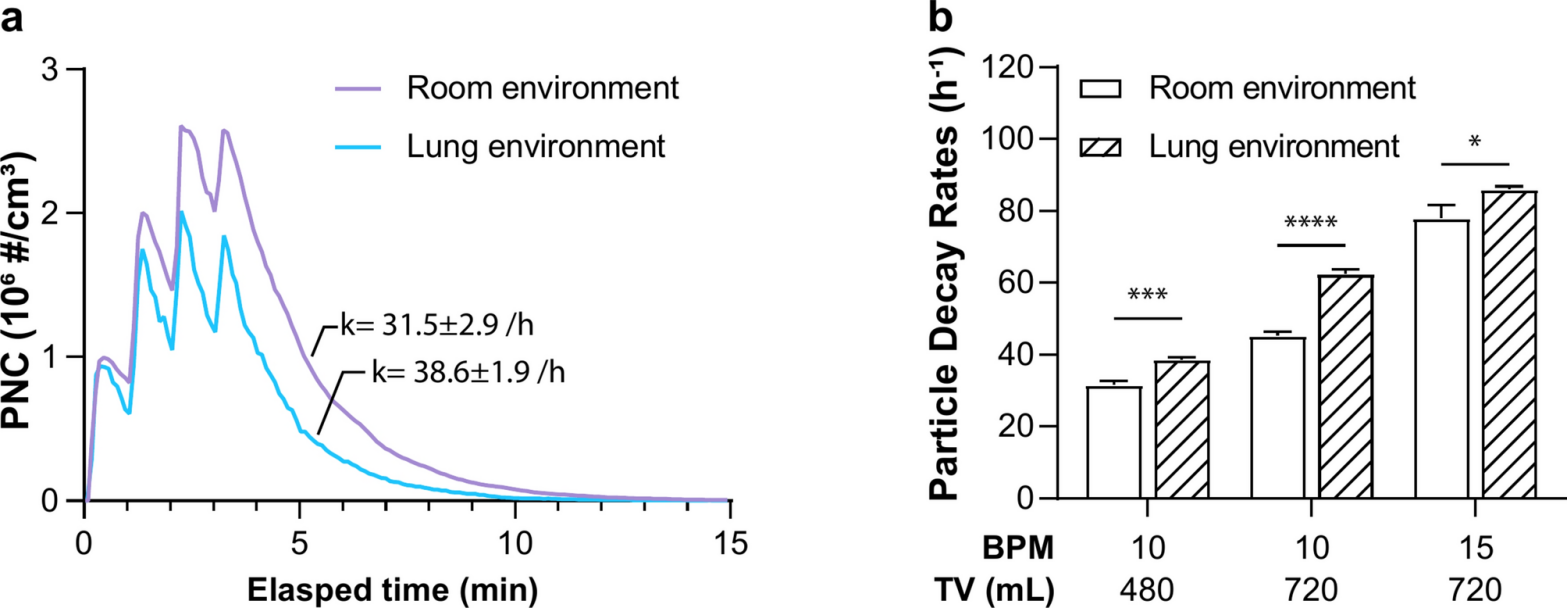
The impact of different environmental conditions on intrapulmonary exposure of e-cig aerosols in a typical vaping session consisting of a 4-puff cycle and the decay (15 min). (**a)** Real-time PNCs within the artificial lung chamber when set up to emulate a standard room environment (38 ± 3% RH; 25 ± 0.5 °C) versus the simulated lung environment (88 ± 5% RH; 37 ± 0.5 °C) under the normal ventilated condition (10 BPM; 480 mL/breath). The PNC curves are representative examples for each experimental condition from triplicate experiments, and the labeled decay rates (k) are presented in 95% CI from the triplicate experiments; (**b)** The comparison of particle decay rates between the room environment and the simulated lung environment under various ventilation conditions. The particle decay rates were obtained from triplicate experiments for each condition. The error bars stand for standard deviations. Significant differences in decay rates between room environment and lung environment at different ventilation patterns were analyzed via t-test (*: p < 0.05, **: p < 0.01, ***: p < 0.001, ****: p < 0.0001).

As air enters the upper respiratory tract it is rapidly warmed and humidified. We identified a significant impact on PNCs when the ventilated lung model was set to simulate the lung environment (88 ± 5% RH, 37 ± 0.5°C) as compared to a room environment (38 ± 3% RH, 25 ± 0.5°C; Fig. 3a). When measured under a resting breathing pattern (10 BPM, 480 mL/breath), the higher humidity and temperature associated with the lung environment reduced peak PNCs by 28% (1.87 ± 0.37 × 10^6^ #/cm^3^, 95% CI) and increased the particle decay rate from 31.5 ± 2.9 to 38.6 ± 1.9 h^−1^ (95% CI). A similar increase in the particle decay rates was observed under all breathing patterns when changing the temperature and RH from the room environment to simulated lung environment (Fig. 3b). It appears that the warm and humid lung environment reduced the condensation formation of e-cig particles from vaporized e-liquids and prompted the coagulation scavenging of inhaled e-cig particles, collectively leading to decreased PNCs^26,27^.

### Particle size distributions

**Fig. 4:**
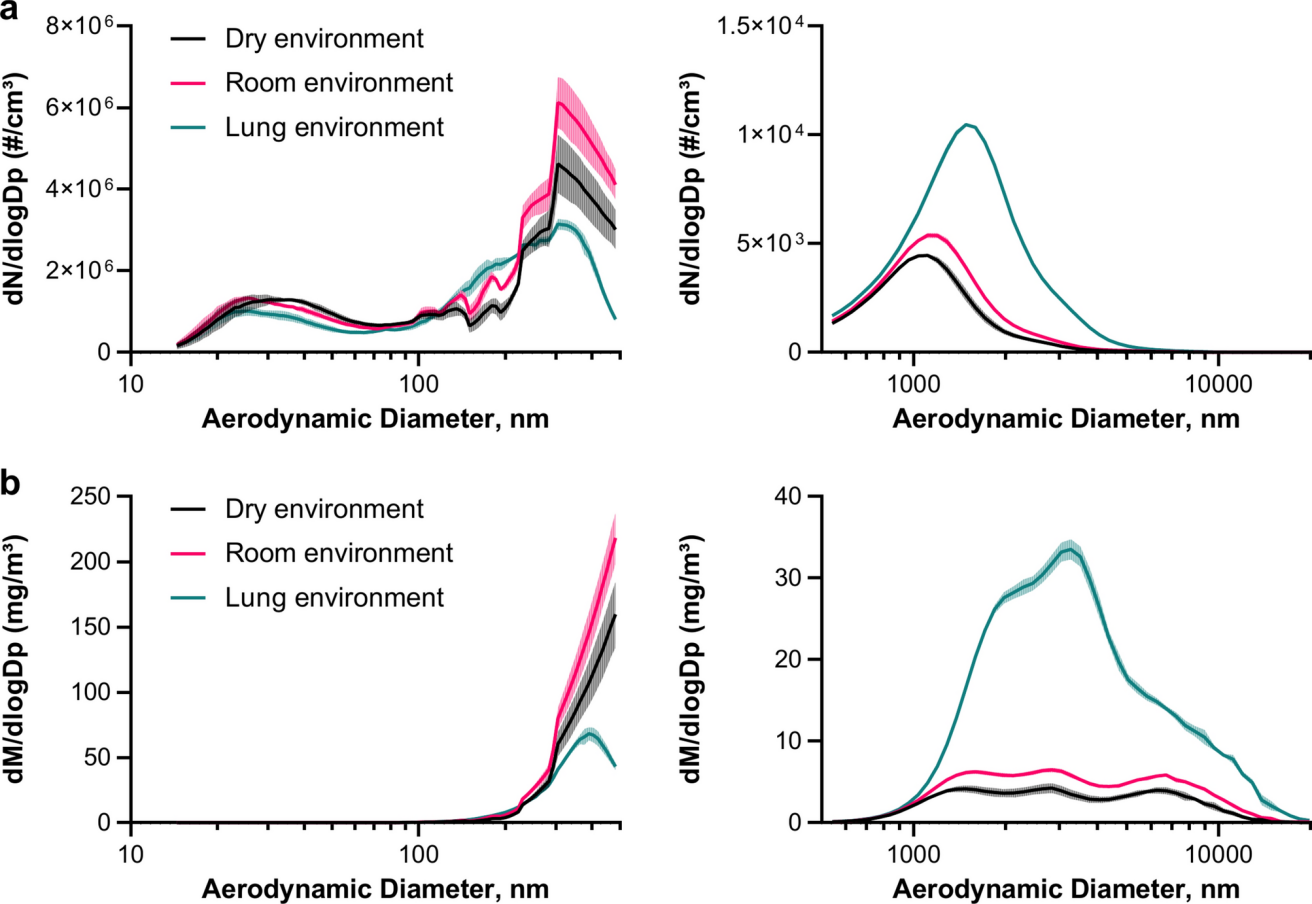
Impact of three different environmental conditions including a dry environment (3 ± 3% RH, 25 ± 0.5°C), a room environment (38 ± 3% RH; 25 ± 0.5°C) and a simulated lung environment (88 ± 5% RH; 37 ± 0.5°C) on e-cig particle size distributions. (**a**) number-based and (**b**) mass-based particle size distributions of e-cig aerosols within the ventilated lung chamber after vaping 4 puffs, measured using a Scanning Mobility Particle Sizer (SMPS) and an Aerodynamic Particle Sizer (APS). The data are shown in separate plots for the measurement size ranges of the SMPS (left) and APS (right). The mean measurements from triplicate experiments for each environmental condition are depicted in lines and the standard deviations are indicated by shaded areas.

Particle size distributions of e-cig aerosols within the lung chamber were measured immediately after the first and last puff of the 4-puff vaping cycle and at 5 min thereafter during the decay period. Figure 4 shows the e-cig particle size distributions in the ventilated lung model at the completion of the 4-puff vaping cycle under dry, room, and simulated lung environmental conditions. Regardless of the environmental conditions, a clear trimodal particle number-based size distribution was observed. This distribution comprised two predominant modes: submicron (~ 300 nm, mode diameter) and ultrafine (25–35 nm, mode diameter) particles, as captured by the SMPS. Additionally, there was a tertiary mode of micron particles (1.0–1.5 μm, mode diameter) captured by the APS, present at number concentrations two to three orders of magnitude lower than those of the submicron and ultrafine particles. The particle size distribution data for each aerosol mode are summarized in **Table S1**. The environmental conditions significantly impacted particle size distributions through aerosol transformations. Most notably, as shown in Fig. 4a, more and larger micron-sized particles were observed under the lung environment [4650 ± 86 #/cm^3^, 1.46 ± 0.04 μm (geometric mean)] compared to the room environment [2220 ± 108 #/cm^3^, 1.17 ± 0.02 μm (geometric mean)]. These larger particles captured by the APS are likely formed due to the hygroscopic growth of smaller particles by absorbing water vapor, which is particularly fostered by this warm and humid environment. This observation is consistent with prior studies showing that exhaled e-cig aerosols have larger-sized particle distributions than the mainstream aerosols emitted directly from e-cig devices^41,42^. Consequently, the increase in micron particle number concentrations and size leads to a significant mass increase of the micron mode, as shown in Fig. 4b and **Table S1**. As stated in previous modeling studies on e-cig particles, larger particles (> 0.5 µm) tend to grow more significantly than smaller particles resulting in mass gain^10,27^. However, as the SMPS in this study used room air for the sheath flow, it did not capture any potential hygroscopic growth for particles within its size range. In contrast to the aerosol growth in the lung environment, the dry environment caused fewer and smaller micron-sized particles [1770 ± 138 #/cm^3^ (number concentrations), 1.11 ± 0.06 μm (geometric mean)] compared to the room environment, possible due to evaporation.

**Fig. 5:**
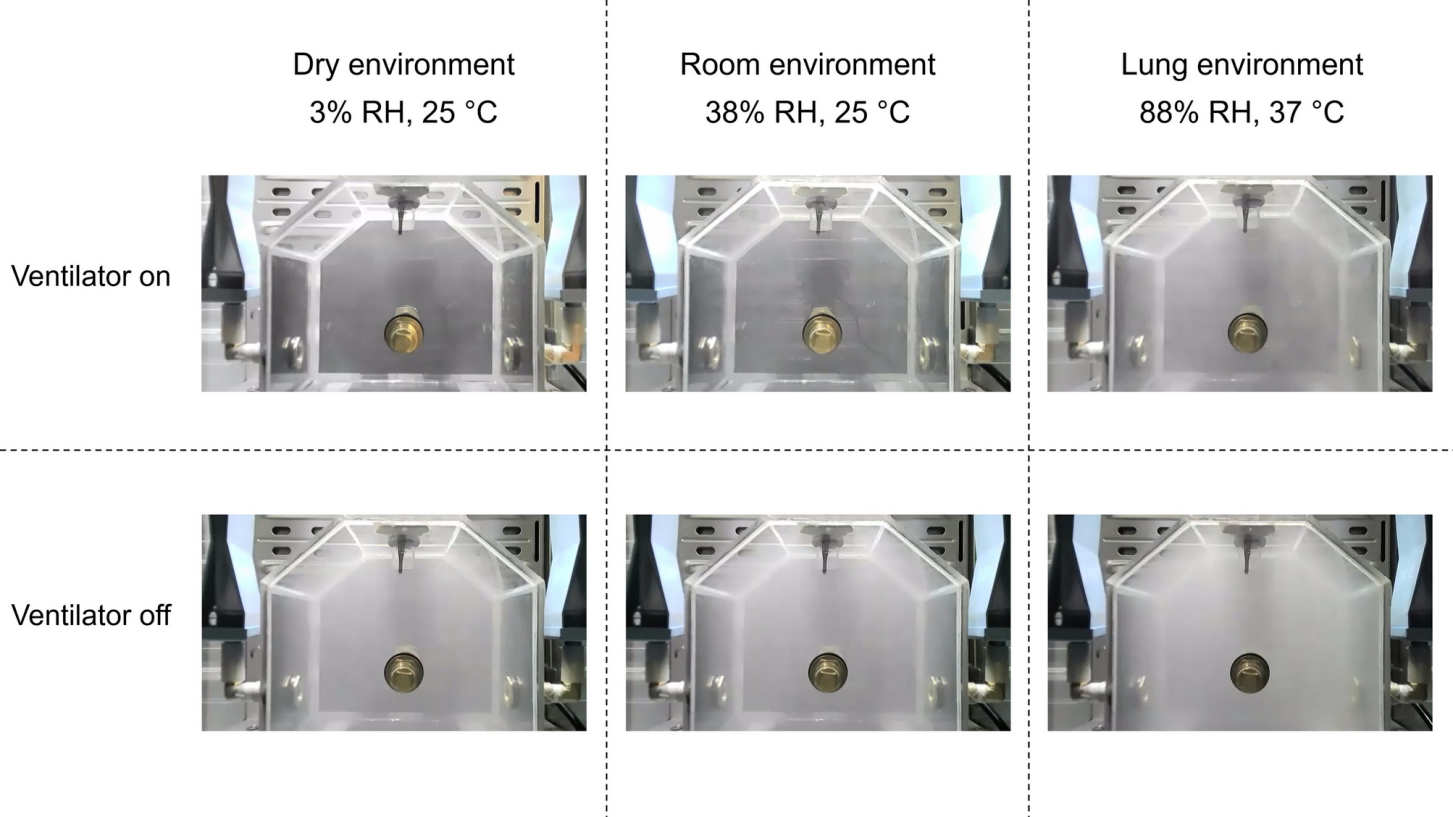
Visualization of e-cig aerosols within the artificial lung chamber under different ventilation and environmental conditions. Images of the artificial lung exposure chamber were captured immediately after the 4th puff with either the ventilator turned on (top row: 10 BPM; 480 mL/breath) or under static conditions (bottom row) and under three different environmental conditions.

Consistent with the real-time PNC data, the time-resolved particle size distributions from the first puff to 5 min after the fourth puff confirmed that ventilation (**Fig. S2 a, b**) resulted in a dramatically faster particle decay rate compared to static conditions (**Fig. S2 c, d**). Ongoing ventilation removed inhaled e-cig particles by 97 ± 1.3% (95% CI) in number concentrations and 89 ± 2% (95% CI) in mass concentrations from the artificial lung chamber within 5 min (**Table S2**). As shown in **Fig. S2 a, c**, during the repetitive vaping, we observed an upward shift in particle size for ultrafine particles over time (from the first to the fourth puff), in both ventilated and static lung models. This is possibly due to the coagulation of ultrafine particles when accumulated in the lung chamber by repetitive puffing. However, the size of ultrafine mode particles (i.e., geometric mean) observed in the ventilated lung models was smaller than that in the static lung models (**Table S2**), suggesting the effects of continuous dilution from ventilation on particle coagulation in the lung chamber. In both the ventilated and static lung models, the warm and humid lung environment promoted hygroscopic growth, forming larger micron particles. Nevertheless, the static condition facilitates the continuous formation of these larger particles throughout the repetitive vaping cycle and retains them afterward due to the lack of dilution. The visual effects of the different environmental conditions and ventilation on the density of the aerosol cloud within the artificial lung was captured by video recording. Still images obtained immediately after inhalation of the fourth puff are compared in Fig. 5.

## Discussions

Human lungs act as the primary portal for exposure to e-cig aerosols from both active use and environmental sources. Understanding the characteristics and behavior of e-cig aerosols within the lung is crucial to understanding vaping-related lung exposures^8–10^. Most studies on e-cigs have focused on aerosol emission characteristics, which are studied under conditions substantially different from those within the lung^13–16^. In this study, we employed a ventilated artificial lung model that replicates two key physiological features of human lungs, ventilation (overall lung volumes and breathing patterns) and lung environment (temperature and RH), to study their impacts on the inhaled aerosols.

Our studies illustrate the dynamic nature of the liquid-based e-cig particles as they enter the lung and confirm our concerns that standard laboratory-based characterization is not, by itself, sufficient to understand lung exposures. The first key finding is that PNCs within the ventilated artificial lung, following a 4-puff vaping cycle, can reach peak levels averaging 2.61 ± 0.64 × 10^6^ #/cm^3^ using the puff regime in this study. Even when time-averaged over the 10-min period from first puff through 90% removal of the inhaled particles, the average PNCs can exceed 10^6^ #/cm^3^. These intense vaping-related exposures, occurring repeatedly and frequently, expose the lung to particle concentrations that are orders of magnitude higher than those occurring from environmental sources such as air pollution, where PNCs are usually ≤ 10^4^ #/cm^3^^43,44^. Vaping topography is highly variable and for individuals who inhale larger puff volumes (ranges 35 to 85 mL), have shorter puffing intervals (range 9 to 55 s), and/or inhale greater than 4 puffs in a session (range 10 to 55 puffs), intrapulmonary exposure levels could be substantially higher^36^. Our findings demonstrate that ongoing ventilation plays a key role in modulating lung exposures by serially diluting and eliminating retained aerosols with each subsequent breath of fresh air. Even normal quiet breathing can reduce peak PNC and AUC within the lung chamber by approximately 40% and 70%, respectively, compared to static conditions. Based on these findings, one might expect that individuals sensitive to the irritative effects of e-cig vapor would subconsciously increase respiratory rate and/or tidal volume during their vaping sessions as a mechanism to reduce the magnitude and intensity of exposure. Given the key role of lung volume and physiology on exposure, one would also expect that individuals with smaller lungs or minute ventilations (which vary by age, sex, and ethnicity), and those with altered physiology due to co-existing lung diseases (such as asthma, COPD, restrictive lung diseases), might experience significant differences in aerosol exposure and risk for toxicity. This aspect of inhaled aerosol science warrants additional investigation.

Another key insight from our investigation is that the particle size distributions of inhaled e-cig aerosols are rapidly and significantly changed as they enter the lung environment. All three e-cig aerosol modes produced by the JUUL pod, including ultrafine (25–35 nm), submicron (~ 300 nm), and micron (~ 1–1.5 μm) ranges, are known to be susceptible to aerosol transformations such as condensation, coagulation, and evaporation^45^. The warm and humid lung environment appeared to promote the hygroscopic growth of e-cig aerosols into larger particles, which contributed significantly to the mass fractions of the inhaled e-cig aerosols. It is worthwhile to note that the hygroscopic growth recorded in this study occurred under a simulated lung environment with 88 ± 5% RH. According to Köhler theory, hygroscopic growth would likely be greater in a human lung where the actual RH approaches 99%^46^. These findings align with previous studies suggesting that e-cig particles exhibit high hygroscopicity due to their unique compositions consisting mainly of PG and VG^10,26,27,47^. Since particle size directly impacts the lung deposition of inhaled aerosols leading to the final inhalation exposure dose, these data provide novel insights regarding the physiochemical transformation of e-cig aerosols within human lungs and the resulting lung exposure.

In addition to the insights provided here, the ventilated artificial lung model has the potential for future adaptations and applications. As currently constructed, the model replicates the overall lung volumes, ventilatory characteristics, and environmental physiology of a breathing lung, but lacks the airway and alveolar structures required to emulate the distributive airflow features and particle deposition that are inherent to human lungs^48,49^. Our study shows two predominant e-cig particle modes: submicron (~ 300 nm) and ultrafine (25–35 nm). According to accepted models for lung deposition^49^, submicron particles exhibit a lower deposition fraction, while ultrafine particles are deposited to a greater extent, particularly in the alveolar region. As a result, due to the absence of airway and alveolar structures, the observed PNC levels are likely overestimated, and particle decay rates underestimated. Additionally, without conducting airways to distribute inhaled airflow, aerosol mixing occurs more rapidly in our model than in human lungs. Nonetheless, our model represents an important advancement in understanding the role of ventilation and the lung environment in modifying e-cig aerosols exposure. A 3D-printed human airway structure has been developed and is currently undergoing evaluation. This addition will allow us to directly assess the role of airway deposition on time-resolved airway and lung exposure. Additional modeling will be needed to replicate the large surface area associated with the presence of 274–790 million alveoli in the human lung^48,49^ This model was specifically designed as a sterile, self-contained incubation chamber that replicates the conditions within a functioning lung. It therefore provides a unique opportunity to expose primary human bronchial and alveolar air–liquid-interface (ALI) cultures to realistic levels and patterns of inhaled aerosols for studying their biologic impact. The capacity to fully characterize recurring lung exposure conditions, as described here, and to simultaneously investigate their biological effects on human epithelium in the future, is anticipated to provide novel insight into the pathogenesis of exposure-related lung disease. Finally, while the current studies focus on e-cig aerosols, this approach has wide-ranging implications for the study of other inhaled substances, medical aerosols and environmental exposures.

In conclusion, this work introduces a novel in vitro lung model that integrates realistic ventilation, lung physiology and lung environmental conditions for investigating intrapulmonary exposure to e-cig aerosols. Results from this study demonstrate the dynamic intrapulmonary exposure profiles of e-cig aerosols from a typical vaping session and reveal the impact of both ongoing ventilation and the lung environment on the characteristics of inhaled e-cig aerosols. Future research may allow us to estimate particle deposition in various lung regions by integrating modeled airway and alveolar structures and to directly investigate the pathobiology of lung disease by exposing bronchial and alveolar epithelial cultures within the model. There is also an important opportunity to study the impact of vaping topography, lung size and disease states on both exposures and toxicity.

## Supplementary Information

Supplementary Information.

## Data Availability

All data for this study are provided within the manuscript or supplementary information files.
