# Supplementary material for: Ventilation and features of the lung environment dynamically alter modeled intrapulmonary aerosol exposure from inhaled electronic cigarettes
Source: Sci Rep. 2024 Dec 30;14:31683. doi: 10.1038/s41598-024-81066-x (PMC11686258; doi:10.1038/s41598-024-81066-x)
Supplement: Supplementary file 1 — Supplementary Information. [file 41598_2024_81066_MOESM1_ESM.docx]

**Ventilation and features of the lung environment dynamically alter modeled intrapulmonary aerosol exposure from inhaled electronic cigarettes**

**Supporting Information**

Liqiao Li^1†^, Haoxuan Chen^1†^, Yifang Zhu^1^, Airi Harui^2^, and Michael D. Roth^2^*

1. Department of Environmental Health Sciences, Jonathan and Karin Fielding School of Public Health, University of California, Los Angeles, CA 90095-1772, USA

2. Division of Pulmonary and Critical Care, Department of Medicine, David Geffen School of Medicine, University of California, Los Angeles, CA 90095-1690, USA

†Co-first author

*Corresponding author

email: [mroth@mednet.ucla.edu](mailto:mroth@mednet.ucla.edu)


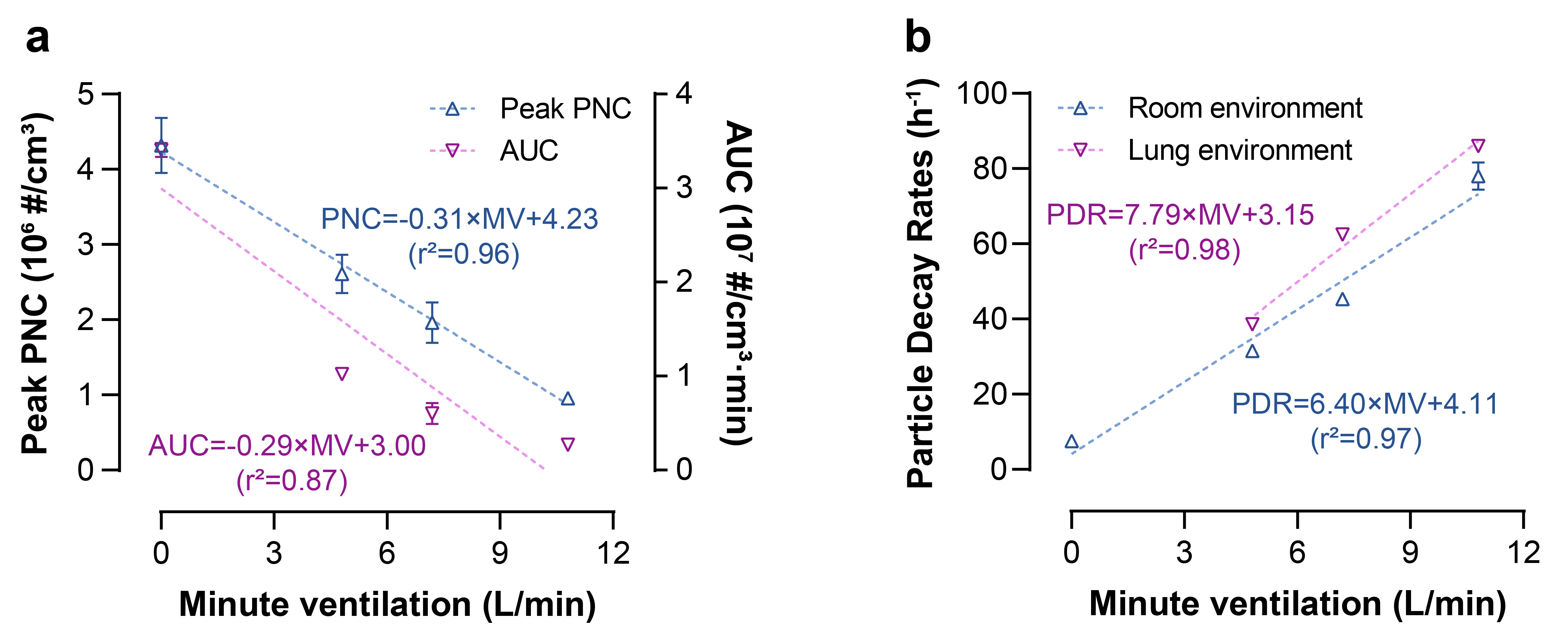


**Figure S1.** The impact of ventilation on intrapulmonary exposure of e-cig aerosols in a typical vaping session consisting of a 4-puff cycle and the decay. **a)** The peak particle number concentrations (PNCs) and the area under the PNC curve (AUC) for the entire vaping session and their correlation to the minute ventilation (MV, respiratory rate × tidal volume); **b)** Particle decay rates (PDRs) during the decay period after a 4-puff vaping cycle under the room and lung environments and their correlation with the MV. The linear regression was fitted.


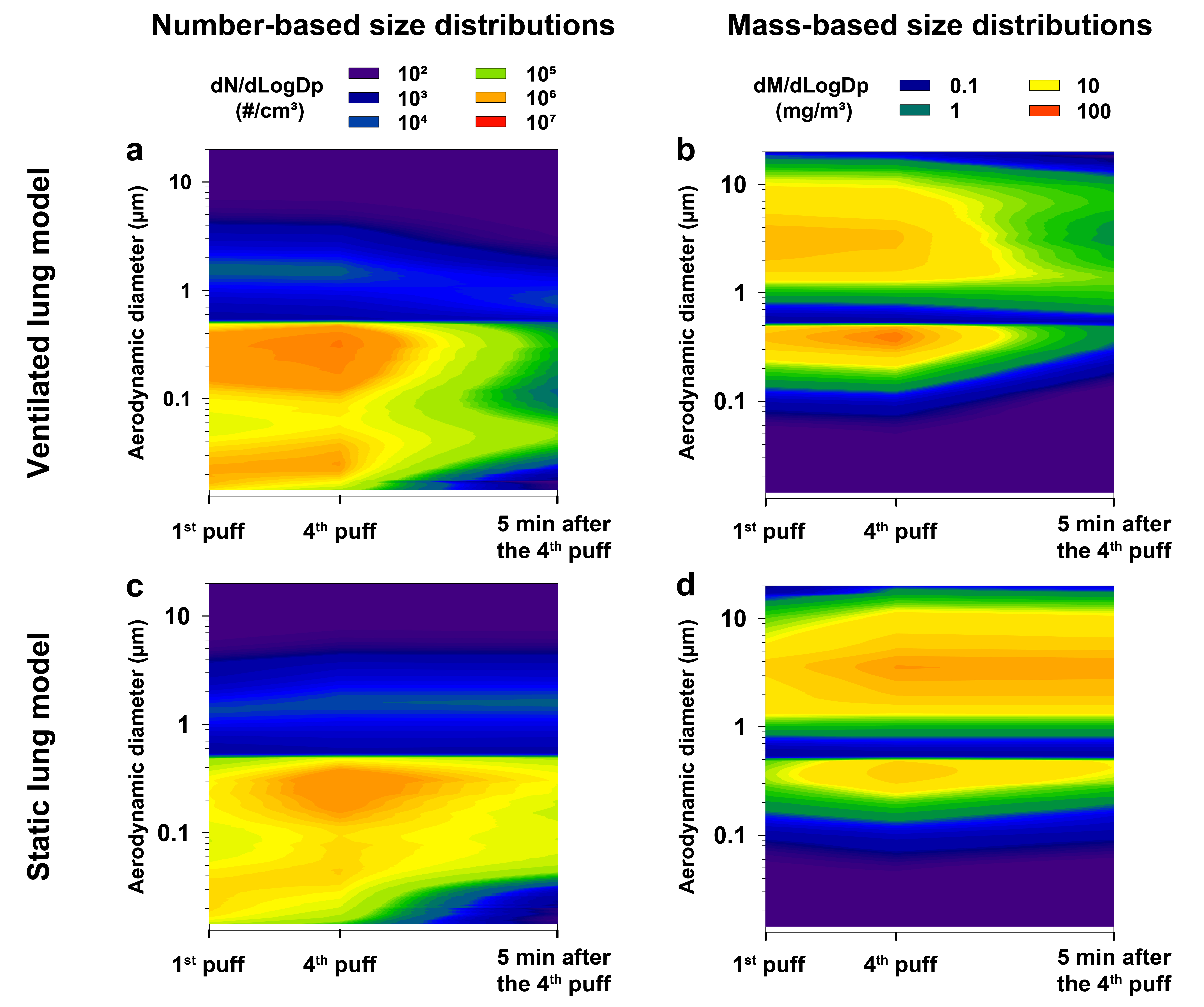


**Figure S2.** Time-resolved e-cig particle size distributions over the simulated 4-puff vaping session in ventilated and static lung models under lung environment. (**a, c**) Number-based and (**b, d**) Mass-based particle size distributions of e-cig aerosols within the lung chamber, measured using a Scanning Mobility Particle Sizer (SMPS) and an Aerodynamic Particle Sizer (APS) at discrete time points in both the ventilated (10 BPM; 480 mL/breath) (**a, b**) and static lung models (**c, d**), under lung environmental conditions. The vertical axes represent particle size on a logarithmic scale, the horizontal axes represent elapsed time in minutes during the decay, and the color scale indicates particle number concentration at a specific time and particle diameter. The figures represent average measurements from triplicate experiments for each experimental condition. Linear interpolation was used to create a continuous pattern of change between the discrete data points.

**Table S1.** Statistics of e-cig aerosols within the ventilated lung chamber after vaping 4 puffs under different environmental conditions. The aerosol modes were fitted with Lognormal or Rosin-Rammler distributions, whichever provided the best fit. The data are presented as mean and standard deviation from triplicate experiments. The total number and mass concentrations are presented in #/cm^3^ and μg/m^3^, respectively.

| **Num-based size distribution** | | **Ventilated lung** | | | | | | | | | | | |
| --- | --- | --- | --- | --- | --- | --- | --- | --- | --- | --- | --- | --- | --- |
|  |  | **Dry condition** | | | | **Room condition** | | | | **Lung condition** | | | |
|  |  | **Number particle size** | | **Mass particle size** | | **Number particle size** | | **Mass particle size** | | **Number particle size** | | **Mass particle size** | |
|  |  | **Mean** | **Std** | **Mean** | **Std** | **Mean** | **Std** | **Mean** | **Std** | **Mean** | **Std** | **Mean** | **Std** |
| **SMPS** | **Median (nm)** | 232.67 | 14.74 | 390.67 | 1.53 | 255.00 | 12.29 | 392.00 | 1.00 | 193.33 | 12.66 | 358.00 | 2.65 |
|  | **Mean (nm)** | 217.33 | 6.35 | 380.33 | 2.08 | 235.67 | 8.14 | 381.33 | 0.58 | 197.33 | 9.61 | 349.67 | 2.31 |
|  | **Geo. Mean (nm)** | 147.33 | 4.51 | 371.33 | 2.08 | 167.33 | 9.45 | 372.67 | 1.15 | 140.67 | 10.50 | 338.00 | 3.46 |
|  | **Mode (nm)** | 304.00 | 0.00 | 482.00 | 0.00 | 304.00 | 0.00 | 482.00 | 0.00 | 304.00 | 0.00 | 390.00 | 0.00 |
|  | **Geo. Std Dev.** | 2.78 | 0.09 | 1.27 | 0.01 | 2.68 | 0.08 | 1.26 | 0.01 | 2.59 | 0.08 | 1.32 | 0.01 |
|  | **Total Conc.** | 2.34E+06 | 2.26E+05 | 2.71E+04 | 3.98E+03 | 2.85E+06 | 1.30E+05 | 3.66E+04 | 3.55E+03 | 2.00E+06 | 7.23E+04 | 1.65E+04 | 9.02E+02 |
| **APS** | **Median (nm)** | 1074.00 | 23.90 | 2874.00 | 67.44 | 1140.67 | 7.51 | 3027.00 | 9.00 | 1467.00 | 16.70 | 3138.67 | 28.75 |
|  | **Mean (nm)** | 1213.33 | 29.57 | 3864.67 | 85.42 | 1289.67 | 7.77 | 4075.33 | 28.01 | 1630.00 | 17.09 | 4011.33 | 40.41 |
|  | **Geo. Mean (nm)** | 1105.00 | 23.90 | 2992.67 | 76.49 | 1168.67 | 6.66 | 3167.33 | 10.60 | 1464.33 | 14.64 | 3300.33 | 33.86 |
|  | **Mode (nm)** | 1088.33 | 44.46 | 2353.33 | 841.20 | 1141.67 | 47.92 | 2839.00 | 0.00 | 1486.00 | 0.00 | 3278.00 | 0.00 |
|  | **Geo. Std Dev.** | 1.50 | 0.01 | 2.06 | 0.02 | 1.52 | 0.00 | 2.05 | 0.01 | 1.58 | 0.01 | 1.84 | 0.01 |
|  | **Total Conc.** | 1.77E+03 | 55.68 | 3.80E+03 | 4.36E+02 | 2.22E+03 | 43.59 | 5.74E+03 | 1.93E+02 | 4.65E+03 | 34.64 | 2.11E+04 | 6.56E+02 |
| **Mode 1  (Lognormal)** | **Median (nm)** | 37.71 | 1.65 | 125.06 | 54.17 | 32.75 | 3.75 | 78.63 | 25.41 | 28.74 | 0.95 | 48.38 | 0.43 |
|  | **Geo. Mean (nm)** | 37.71 | 1.65 | - | - | 32.75 | 3.75 | - | - | 28.74 | 0.95 | _ | - |
|  | **Geo. Std. Dev.** | 1.85 | 0.22 | - | - | 1.70 | 0.10 | - | - | 1.52 | 0.03 | _ | - |
|  | **Geo. Mode (nm)** | 37.71 | 1.65 | 125.06 | 54.17 | 32.75 | 3.75 | 78.63 | 25.41 | 28.74 | 0.95 | 48.38 | 0.43 |
|  | **Total Conc.** | 7.87E+05 | 1.22E+05 | 1.65E+02 | 1.21E+02 | 6.73E+05 | 2.54E+04 | 58.19 | 40.09 | 4.36E+05 | 6.90E+04 | 13.58 | 1.70 |
| **Mode 2  (Rosin_**  **Rammler)** | **Median (nm)** | 308.80 | 4.20 | - | - | 317.45 | 6.17 | - | - | 235.83 | 6.94 | - | - |
|  | **Geo. Mean (nm)** | 277.92 | 3.78 | - | - | 285.70 | 5.55 | - | - | 212.25 | 6.24 | - | - |
|  | **Geo. Std. Dev.** | - | - | - | - | - | - | - | - | - | - | - | - |
|  | **Geo. Mode (nm)** | 370.91 | 5.04 | 586.45 | 7.96 | 381.29 | 7.41 | 602.88 | 11.71 | 283.26 | 8.33 | 447.88 | 13.17 |
|  | **Total Conc.** | 1.70E+06 | 1.74E+05 | 6.96E+04 | 1.01E+04 | 2.51E+06 | 4.93E+04 | 1.12E+05 | 8.08E+03 | 1.62E+06 | 3.79E+04 | 2.96E+04 | 2.41E+03 |
| **Mode 3  (Lognormal)** | **Median (nm)** | 1168.78 | 29.88 | 1375.02 | 47.20 | 1103.05 | 8.16 | 1940.06 | 22.44 | 1437.28 | 16.31 | 2786.44 | 37.03 |
|  | **Geo. Mean (nm)** | 1168.78 | 29.88 | - | - | 1103.05 | 8.16 | - | - | 1437.28 | 16.31 | - | - |
|  | **Geo. Std. Dev.** | 1.26 | 0.01 | - | - | 1.54 | 0.01 | - | - | 1.60 | 0.00 | - | - |
|  | **Geo. Mode (nm)** | 1168.78 | 29.88 | 1375.02 | 47.20 | 1103.05 | 8.16 | 1940.06 | 22.44 | 1437.28 | 16.31 | 2786.44 | 37.03 |
|  | **Total Conc.** | 1.26E+03 | 36.06 | 1.55E+03 | 1.87E+02 | 2.27E+03 | 40.41 | 4.27E+03 | 1.83E+02 | 4.65E+03 | 41.63 | 2.25E+04 | 7.77E+02 |

Abbreviations: StD: standard deviation, Geo: Geometric, Dev: Deviation, Conc.: concentration.

**Table S2.** Statistics of e-cig aerosols within the ventilated and static lung chambers under the lung environment that were measured at discrete times (1^st^ puff, 4^th^ puff, and 5 minutes after the 4^th^ puff) during a 4-puff vaping session. The aerosol modes were fitted with Lognormal or Rosin-Rammler distributions, whichever provided the best fit. The data are presented as mean and standard deviation from triplicate experiments. The total number and mass concentrations are presented in #/cm^3^ and μg/m^3^, respectively.

| **Num-based size distribution** | | | **Ventilated lung model** | | | | **Static lung model** | | | |
| --- | --- | --- | --- | --- | --- | --- | --- | --- | --- | --- |
|  |  |  | **Number particle size** | | **Mass particle size** | | **Number particle size** | | **Mass particle size** | |
|  |  |  | **Mean** | **Std** | **Mean** | **Std** | **Mean** | **Std** | **Mean** | **Std** |
| **1st Puff** | **SMPS** | **Median (nm)** | 153.67 | 5.51 | 351.00 | 4.58 | 59.67 | 13.32 | 330.67 | 8.50 |
|  |  | **Mean (nm)** | 167.00 | 3.61 | 343.33 | 4.04 | 112.00 | 8.66 | 325.33 | 6.43 |
|  |  | **Geo. Mean (nm)** | 107.33 | 3.21 | 330.67 | 4.16 | 70.00 | 7.81 | 309.67 | 5.86 |
|  |  | **Mode (nm)** | 279.00 | 43.30 | 399.33 | 8.08 | 30.33 | 8.33 | 376.67 | 27.01 |
|  |  | **Geo. Std Dev.** | 2.92 | 0.03 | 1.34 | 0.01 | 2.70 | 0.06 | 1.41 | 0.01 |
|  |  | **Total Conc.** | 1.30E+06 | 1.27E+05 | 8.36E+03 | 4.82E+02 | 6.90E+05 | 1.99E+05 | 2.24E+03 | 7.18E+02 |
|  | **APS** | **Median (nm)** | 1525.33 | 20.82 | 3246.67 | 14.64 | 1368.00 | 18.73 | 3109.33 | 296.47 |
|  |  | **Mean (nm)** | 1692.00 | 21.38 | 4081.67 | 9.71 | 1547.33 | 36.07 | 3739.00 | 218.38 |
|  |  | **Geo. Mean (nm)** | 1522.00 | 19.00 | 3380.67 | 12.10 | 1399.33 | 23.29 | 3115.67 | 199.26 |
|  |  | **Mode (nm)** | 1559.33 | 63.51 | 3359.67 | 141.45 | 1350.00 | 55.43 | 3698.33 | 151.84 |
|  |  | **Geo. Std Dev.** | 1.57 | 0.01 | 1.82 | 0.02 | 1.54 | 0.04 | 1.81 | 0.03 |
|  |  | **Total Conc.** | 4.62E+03 | 86.22 | 2.32E+04 | 1.13E+03 | 3.67E+03 | 4.49E+02 | 1.42E+04 | 2.86E+03 |
|  | **Mode 1**  **(Lognormal)** | **Median (nm)** | 21.84 | 0.43 | 45.81 | 2.58 | 27.89 | 2.99 | 71.37 | 27.23 |
|  |  | **Geo. Mean (nm)** | 21.84 | 0.43 | - | - | 27.89 | 2.99 | - | - |
|  |  | **Geo. Std. Dev.** | 1.65 | 0.02 | - | - | 1.72 | 0.18 | - | - |
|  |  | **Geo. Mode (nm)** | 21.84 | 0.43 | 45.81 | 2.58 | 27.89 | 2.99 | 71.37 | 27.23 |
|  |  | **Total Conc.** | 4.77E+05 | 5.60E+04 | 9.12 | 1.60 | 3.71E+05 | 7.83E+04 | 22.40 | 18.29 |
|  | **Mode 2**  **(Rosin_**  **Rammler)** | **Median (nm)** | 220.20 | 3.88 | - | - | 180.47 | 12.86 | - | - |
|  |  | **Geo. Mean (nm)** | 198.18 | 3.50 | - | - | 162.42 | 11.58 | - | - |
|  |  | **Geo. Std. Dev.** | - | - | - | - | - | - | - | - |
|  |  | **Geo. Mode (nm)** | 264.49 | 4.66 | 418.19 | 7.38 | 216.76 | 15.44 | 342.73 | 24.42 |
|  |  | **Total Conc.** | 9.41E+05 | 7.80E+04 | 1.39E+04 | 5.57E+02 | 3.61E+05 | 1.23E+05 | 2.93E+03 | 1.06E+03 |
|  | **Mode 3**  **(Lognormal)** | **Median (nm)** | 1501.19 | 21.61 | 2845.26 | 52.23 | 1357.72 | 19.62 | 2422.71 | 193.09 |
|  |  | **Geo. Mean (nm)** | 1501.19 | 21.61 | - | - | 1357.72 | 19.62 | - | - |
|  |  | **Geo. Std. Dev.** | 1.59 | 0.01 | - | - | 1.55 | 0.05 | - | - |
|  |  | **Geo. Mode (nm)** | 1501.19 | 21.61 | 2845.26 | 52.23 | 1357.72 | 19.62 | 2422.71 | 193.09 |
|  |  | **Total Conc.** | 4.59E+03 | 85.44 | 2.44E+04 | 1.56E+03 | 3.61E+03 | 4.84E+02 | 1.30E+04 | 2.87E+03 |
| **4th Puff** | **SMPS** | **Median (nm)** | 193.33 | 12.66 | 358.00 | 2.65 | 160.33 | 26.16 | 342.67 | 22.81 |
|  |  | **Mean (nm)** | 197.33 | 9.61 | 349.67 | 2.31 | 174.67 | 18.45 | 337.33 | 19.76 |
|  |  | **Geo. Mean (nm)** | 140.67 | 10.50 | 338.00 | 3.46 | 126.33 | 13.65 | 324.33 | 21.73 |
|  |  | **Mode (nm)** | 304.00 | 0.00 | 390.00 | 0.00 | 279.00 | 43.30 | 373.33 | 46.06 |
|  |  | **Geo. Std Dev.** | 2.59 | 0.08 | 1.32 | 0.01 | 2.43 | 0.31 | 1.35 | 0.04 |
|  |  | **Total Conc.** | 2.00E+06 | 7.23E+04 | 1.65E+04 | 9.02E+02 | 1.20E+06 | 1.35E+05 | 7.67E+03 | 1.33E+03 |
|  | **APS** | **Median (nm)** | 1467.00 | 16.70 | 3138.67 | 28.75 | 1577.00 | 85.81 | 3495.00 | 221.76 |
|  |  | **Mean (nm)** | 1630.00 | 17.09 | 4011.33 | 40.41 | 1776.33 | 106.02 | 4292.33 | 237.53 |
|  |  | **Geo. Mean (nm)** | 1464.33 | 14.64 | 3300.33 | 33.86 | 1576.00 | 82.78 | 3605.00 | 216.34 |
|  |  | **Mode (nm)** | 1486.00 | 0.00 | 3278.00 | 0.00 | 1599.00 | 114.53 | 3441.33 | 141.45 |
|  |  | **Geo. Std Dev.** | 1.58 | 0.01 | 1.84 | 0.01 | 1.62 | 0.03 | 1.79 | 0.05 |
|  |  | **Total Conc.** | 4.65E+03 | 34.64 | 2.11E+04 | 6.56E+02 | 4.75E+03 | 90.18 | 2.91E+04 | 5.91E+03 |
|  | **Mode 1**  **(Lognormal)** | **Median (nm)** | 28.74 | 0.95 | 48.38 | 0.43 | 43.43 | 15.83 | 81.45 | 27.88 |
|  |  | **Geo. Mean (nm)** | 28.74 | 0.95 | - | - | 43.43 | 15.83 | - | - |
|  |  | **Geo. Std. Dev.** | 1.52 | 0.03 | - | - | 1.59 | 0.06 | - | - |
|  |  | **Geo. Mode (nm)** | 28.74 | 0.95 | 48.38 | 0.43 | 43.43 | 15.83 | 81.45 | 27.88 |
|  |  | **Total Conc.** | 4.36E+05 | 6.90E+04 | 13.58 | 1.70 | 3.82E+05 | 1.03E+05 | 69.40 | 83.67 |
|  | **Mode 2**  **(Rosin_**  **Rammler)** | **Median (nm)** | 235.83 | 6.94 | - | - | 222.91 | 23.08 | - | - |
|  |  | **Geo. Mean (nm)** | 212.25 | 6.24 | - | - | 200.63 | 20.77 | - | - |
|  |  | **Geo. Std. Dev.** | - | - | - | - | - | - | - | - |
|  |  | **Geo. Mode (nm)** | 283.26 | 8.33 | 447.88 | 13.17 | 267.75 | 27.73 | 423.35 | 43.84 |
|  |  | **Total Conc.** | 1.62E+06 | 3.79E+04 | 2.96E+04 | 2.41E+03 | 8.44E+05 | 1.76E+04 | 1.32E+04 | 3.75E+03 |
|  | **Mode 3**  **(Lognormal)** | **Median (nm)** | 1437.28 | 16.31 | 2786.44 | 37.03 | 1549.89 | 86.38 | 3300.08 | 349.08 |
|  |  | **Geo. Mean (nm)** | 1437.28 | 16.31 | - | - | 1549.89 | 86.38 | - | - |
|  |  | **Geo. Std. Dev.** | 1.60 | 0.00 | - | - | 1.65 | 0.03 | - | - |
|  |  | **Geo. Mode (nm)** | 1437.28 | 16.31 | 2786.44 | 37.03 | 1549.89 | 86.38 | 3300.08 | 349.08 |
|  |  | **Total Conc.** | 4.65E+03 | 41.63 | 2.25E+04 | 7.77E+02 | 4.77E+03 | 90.74 | 3.37E+04 | 8.32E+03 |
| **5 min after  the 4th puff** | **SMPS** | **Median (nm)** | 69.67 | 4.51 | 368.00 | 6.24 | 174.33 | 40.50 | 356.33 | 22.12 |
|  |  | **Mean (nm)** | 139.67 | 8.33 | 356.33 | 5.86 | 193.00 | 24.02 | 348.33 | 20.74 |
|  |  | **Geo. Mean (nm)** | 95.33 | 5.03 | 342.67 | 6.43 | 155.33 | 12.58 | 334.67 | 25.11 |
|  |  | **Mode (nm)** | 48.00 | 2.65 | 413.33 | 8.08 | 240.33 | 110.27 | 444.33 | 24.34 |
|  |  | **Geo. Std Dev.** | 2.38 | 0.09 | 1.36 | 0.02 | 1.99 | 0.22 | 1.36 | 0.08 |
|  |  | **Total Conc.** | 5.72E+04 | 9.14E+03 | 2.94E+02 | 35.23 | 3.34E+05 | 6.07E+04 | 2.52E+03 | 9.25E+02 |
|  | **APS** | **Median (nm)** | 905.67 | 33.53 | 1985.33 | 5.13 | 1569.33 | 95.70 | 3401.67 | 189.17 |
|  |  | **Mean (nm)** | 1010.00 | 34.00 | 3570.67 | 103.18 | 1758.67 | 115.80 | 4165.00 | 68.43 |
|  |  | **Geo. Mean (nm)** | 945.33 | 31.02 | 2492.00 | 25.24 | 1568.67 | 93.30 | 3501.00 | 135.57 |
|  |  | **Mode (nm)** | 815.67 | 33.49 | 1382.00 | 0.00 | 1599.00 | 114.53 | 3529.00 | 254.05 |
|  |  | **Geo. Std Dev.** | 1.41 | 0.01 | 2.32 | 0.07 | 1.60 | 0.03 | 1.78 | 0.07 |
|  |  | **Total Conc.** | 3.36E+03 | 1.02E+02 | 3.68E+03 | 1.83E+02 | 4.76E+03 | 1.04E+02 | 2.75E+04 | 5.86E+03 |
|  | **Mode 1**  **(Lognormal)** | **Median (nm)** | 48.04 | 2.80 | 62.16 | 3.30 | 73.78 | 24.30 | 92.85 | 40.01 |
|  |  | **Geo. Mean (nm)** | 48.04 | 2.80 | - | - | 73.78 | 24.30 | - | - |
|  |  | **Geo. Std. Dev.** | 1.34 | 0.01 | - | - | 1.30 | 0.08 | - | - |
|  |  | **Geo. Mode (nm)** | 48.04 | 2.80 | 62.16 | 3.30 | 73.78 | 24.30 | 92.85 | 40.01 |
|  |  | **Total Conc.** | 3.06E+04 | 6.07E+03 | 3.09 | 1.15 | 1.29E+05 | 7.50E+04 | 80.47 | 1.11E+02 |
|  | **Mode 2**  **(Rosin_**  **Rammler)** | **Median (nm)** | 241.02 | 16.86 | - | - | 249.86 | 21.08 | - | - |
|  |  | **Geo. Mean (nm)** | 216.92 | 15.17 | - | - | 224.88 | 18.97 | - | - |
|  |  | **Geo. Std. Dev.** | - | - | - | - | - | - | _ | - |
|  |  | **Geo. Mode (nm)** | 289.49 | 20.24 | 457.72 | 32.01 | 300.11 | 25.31 | 474.52 | 40.02 |
|  |  | **Total Conc.** | 2.64E+04 | 2.20E+03 | 5.18E+02 | 1.21E+02 | 2.10E+05 | 5.23E+04 | 4.80E+03 | 2.43E+03 |
|  | **Mode 3**  **(Lognormal)** | **Median (nm)** | 955.26 | 92.55 | 1254.91 | 18.81 | 1546.61 | 98.89 | 3149.10 | 350.05 |
|  |  | **Geo. Mean (nm)** | 955.26 | 92.55 | - | - | 1546.61 | 98.89 | - | - |
|  |  | **Geo. Std. Dev.** | 1.35 | 0.08 | - | - | 1.63 | 0.03 | - | - |
|  |  | **Geo. Mode (nm)** | 955.26 | 92.55 | 1254.91 | 18.81 | 1546.61 | 98.89 | 3149.10 | 350.05 |
|  |  | **Total Conc.** | 2.35E+03 | 1.18E+03 | 1.78E+03 | 6.39E+02 | 4.75E+03 | 1.04E+02 | 3.13E+04 | 8.86E+03 |

Abbreviations: StD: standard deviation, Geo: Geometric, Dev: Deviation, Conc.: concentration.
